# Detection of Astrovirus in a Cow with Neurological Signs by Nanopore Technology, Italy

**DOI:** 10.3390/v12050530

**Published:** 2020-05-11

**Authors:** Guendalina Zaccaria, Alessio Lorusso, Melanie M. Hierweger, Daniela Malatesta, Sabrina VP Defourny, Franco Ruggeri, Cesare Cammà, Pasquale Ricci, Marco Di Domenico, Antonio Rinaldi, Nicola Decaro, Nicola D’Alterio, Antonio Petrini, Torsten Seuberlich, Maurilia Marcacci

**Affiliations:** 1 Istituto Zooprofilattico Sperimentale dell’Abruzzo e Molise, 64100 Teramo, Italy; guendalina.zaccaria@gmail.com (G.Z.); d.malatesta@izs.it (D.M.); defournysabrina6@gmail.com (S.V.D.); c.camma@izs.it (C.C.); m.didomenico@izs.it (M.D.D.); a.rinaldi@izs.it (A.R.); n.dalterio@izs.it (N.D.); a.petrini@izs.it (A.P.); m.marcacci@izs.it (M.M.); 2 NeuroCenter, Department of Clinical Research and Veterinary Public Health, University of Bern, 3012 Bern, Switzerland; melanie.hierweger@vetsuisse.unibe.ch; 3 Graduate School for Cellular and Biomedical Sciences, University of Bern, 3012 Bern, Switzerland; torsten.seuberlich@vetsuisse.unibe.ch; 4 Unità Operativa Complessa Servizio di Sanità Animale, ASL Pescara, 65100 Pescara, Italy; sanita.pe@ausl.pe.it (F.R.); pricci4@virgilio.it (P.R.); 5 Dipartimento di Medicina Veterinaria, Università degli Studi di Bari, 70010 Valenzano, Italy; nicola.decaro@uniba.it

**Keywords:** bovine astrovirus, nanopore technology, brain, neurological signs, cattle, phylogeny, ISH, Italy

## Abstract

In this study, starting from nucleic acids purified from the brain tissue, Nanopore technology was used to identify the etiological agent of severe neurological signs observed in a cow which was immediately slaughtered. Histological examination revealed acute non-suppurative encephalomyelitis affecting the brainstem, cerebrum, cerebellum, and medulla oblongata, while by using PCR-based assays, the nucleic acids of major agents for neurological signs were not detected. By using Nanopore technology, 151 sequence reads were assigned to Bovine Astrovirus (BoAstV). Real-time RT-PCR and in situ hybridization (ISH) confirmed the presence of viral RNA in the brain. Moreover, using the combination of fluorescent ISH and immunofluorescence (IF) techniques, it was possible to detect BoAstV RNA and antigens in the same cells, suggesting the active replication of the virus in infected neurons. The nearly whole genome of the occurring strain (BoAstV PE3373/2019/Italy), obtained by Illumina NextSeq 500, showed the highest nucleotide sequence identity (94.11%) with BoAstV CH13/NeuroS1 26,730 strain, an encephalitis-associated bovine astrovirus. Here, we provide further evidence of the role of AstV as a neurotropic agent. Considering that in a high proportion of non-suppurative encephalitis cases, which are mostly indicative of a viral infection, the etiologic agent remains unknown, our result underscores the value and versatility of Nanopore technology for a rapid diagnosis when the PCR-based algorithm gives negative results.

## 1. Introduction

Astroviruses (AstVs) are small, about 28–30 nm in diameter, and are non-enveloped, positive-sense, single-stranded RNA viruses with a genome of 6.4–7.3 kb [1]. The *Astroviridae* family comprises two genera, the *Mamastrovirus* and *Avastrovirus*, which are frequently associated with cases of enteric infection in young individuals in a wide plethora of animal species, including domestic, synanthropic, and wild mammals and birds [2]. The name “astrovirus” derives from the star-like appearance of the virions detectable by negative stain electron microscopy, although it is pH-dependent and may only be present in a small part of the virus population [3]. The viral genome is poly-adenylated and includes three partially overlapping open-reading frames (ORF), namely, ORF1a, ORF1b, and ORF2, flanked by untranslated regions (UTR) at the 5’ and 3’ termini [4]. ORF1a and ORF1b encode for polyproteins that are translated into the precursor non-structural polyproteins (nsp) 1a and 1ab via a ribosomal frame shift mechanism, a combination of a slippery (A)6C sequence and downstream structural hairpin. Post-translational cleavage generates a protease, an RNA-dependent RNA polymerase, and a number of proteins of unknown function [5,6]. ORF2 is expressed from a subgenomic RNA and encodes for the viral capsid protein [7].

AstV (genus *Mamastrovirus*) enteric infection is a leading cause of gastroenteritis in children worldwide [8]. Since the first detection of the human astrovirus (HuAstV) in children with diarrhea in 1975 [9], a wide variety of AstVs has been reported in several animal species, mainly associated with enteric diseases, but several asymptomatic infections were also demonstrated [2]. Regardless, most recently, several novel strains were associated to cases of fatal encephalitis both in humans [10,11,12,13,14] and in animals. Encephalitis-associated AstVs have been reported in cattle [15,16,17,18,19], minks [20], muskox [21], and sheep [22,23]. In a high proportion of non-suppurative encephalitis cases (which is mostly indicative of a viral infection [24,25]), the etiologic agent remains unknown [26]. Pathogen identification is usually performed by direct diagnostic tests, which normally include amplification of target nucleic acids by PCR-based assays. Although these approaches are highly specific, they suffer a number of limitations, including the difficulties of testing for the plethora of pathogens that might have caused the disease and their inability to detect new or unexpected pathogens. In these cases, a metagenomic approach can provide a powerful and unbiased tool to identify a pathogen agent [27]. In this manuscript, we describe the identification by next-generation sequencing (NGS) and characterization of a BoAstV strain from the brain tissue of a cow.

## 2. Materials and Methods

### 2.1. Case Description

On 10 May 2019, a 16-month-old Marchigiana (an Italian breed of beef cattle) heifer from a farm consisting of 280 cows located in the Abruzzi region (central Italy) showed blindness and depressed sensorium. The animal was born in the farm where the replacement heifers are mostly retained from the calf crop and not purchased. The case was promptly reported to the Local Veterinary Services. During clinical examination, the animal showed no fever, drooling, or nasal discharge, with normal cardiac and respiratory frequencies. The cow was lying on the ground and unable to stand correctly, with the head turned to the side. During an attempt to get up and walk, the animal was able to stand just on the front fetlock joints, keeping the legs crossed. This animal was the only cow on the farm showing the described clinical signs. The animal was promptly slaughtered, and sample collection was performed at the slaughterhouse.

### 2.2. Samples Collection and Diagnostic Investigation

Samples from different regions of the brain were collected and sent to the Istituto Zooprofilattico Sperimentale dell’ Abruzzo e del Molise (IZSAM) for diagnostic investigations. For histological examination, brain tissues were fixed in 10% neutral buffered formalin and embedded in paraffin wax. Sections (4 µm) were stained with haematoxylin and eosin (HE) and observed using a Leica DMR optical microscope. A brain tissue of a healthy calf regularly slaughtered was used as control. Viral nucleic acid purification was performed using the High Pure Viral Nucleic Acid Kit (Roche Life Science, Basel, Switzerland), following the manufacturer’s instructions. Briefly, 30 mg of brain tissue was homogenized in 1 ml of phosphate-buffered saline (PBS) using a TissueLyser II (Qiagen, Hilden, Germany), and then centrifuged. Two hundred microliters (µL) of the supernatant were used for nucleic acid extraction. The sample was tested by molecular assays for the presence of RNA/DNA of several neurotropic viruses, including pestiviruses (ID Gene™ BVD/BD Triplex (IDBVD) kit, IDvet Genetics), bovine herpesvirus 1 (BoHV-1, VetMax IBR/BHV-1 Reagents + VetMax-Plus qPCR Master Mix—Applied Biosystem, Waltham, MA, USA), ovine gammaherpesvirus type 2 (OvHV-2, TaqMan Universal PCR Master Mix—Applied Biosystem, Thermo Fisher Scientific, Waltham, MA, USA), West Nile virus (WNV, [28]), and Usutu virus (USUV, [29]). For the detection of bacteria and protozoan parasites, 25 mg of brain tissue was added directly to a cartridge of a Maxwell 16 Tissue DNA Purification kit (Promega, Madison WI) for homogenization and DNA extraction steps. Purified DNA was then tested for the presence of *Chlamydia psittaci* (Primerdesign Ltd Chlamydia psittaci, gidA gene—genesig Advanced Kit, Rownhams, UK), *Listeria spp.* (Primerdesign Ltd Lysteria, Invasion-associated Protein p60 (iap) gene—genesig Advanced Kit, Rownhams, UK), *Neospora caninum* (Primerdesign Ltd Neospora caninum, Nc5 marker genomic sequence—genesig Advanced Kit, Rownhams, UK), and *Toxoplasma gondii* (Primerdesign Ltd Toxoplasma gondii, Repeat region—genesig Advanced Kit, Rownhams, UK). Brain tissue samples were also used for the standard procedure for aerobic bacterial isolation and histopathology. 

### 2.3. Shotgun Metagenomics by MinION

Two hundred µL of the brain homogenate were enrolled for nucleic acid purification through the High Pure Viral Nucleic Acid Kit (Roche, Basel, Switzerland) and used for metagenomic analysis. Nucleic acid elution was divided into two aliquots to perform RNA and DNA sequencing, as previously described by our group [27,30]. After Turbo DNAse incubation, RNA was processed by means of the Sequence Independent Single Primer Amplification (SISPA) method to obtain cDNA [31,32]. DNA and amplified cDNA were quantified by Qubit dsDNA HS assay (Thermo Fisher Scientific, Waltham, MA, USA) and used for library preparation by low-input genomic DNA by PCR Barcoding (SQK-LWB001, Oxford Nanopore Technologies, Oxford, UK), following the manufacturer’s guidelines. Sequencing adapters were added prior to library loading on the flow cell MIN106, R9 version (Oxford Nanopore Technologies, Oxford, UK). All purification steps were carried out using AMPure XP beads (Agencourt, Beckman Coulter Brea, CA, USA) according to the SQK-LWB001 sequencing protocol. For sequencing, the NC_48hr_sequencing_FLO-MIN106_SQK-LBW001 program was run on MinKNOW Software v.1.4.2. 

### 2.4. Real-Time RT-qPCR_BoAstV_

A real-time RT-PCR using specific primers for BoAstV [33] was performed using RNA purified from the brain tissue sample. More specifically, the 25 μL reaction volume contained 5 μL of total purified RNA, 12.5 μL of 2× Reaction Mix, 0.5 μL of SuperScript™ III RT/Platinum^®^ Taq High Fidelity Enzyme Mix, 0.05 μL of ROX Reference dye, 1 μL of MgSO_4_ (SuperScript One-Step RT-PCR System with Platinum Taq DNA Polymerase, Invitrogen, Thermo Fisher Scientific, Waltham, MA, USA), a final concentration of 600 nM of both forward (CH13_488Fq) and reverse (CH13_695Rq) primers, and 300 nM of probe (CH13_609Pq) and nuclease-free water up to the final volume. The thermal profile consisted of a single cycle of reverse transcription at 50 °C for 15 min, followed by a denaturation step at 95 °C for 2 min for reverse transcriptase inactivation and DNA polymerase activation. The amplification of cDNA was performed by 45 cycles, including denaturation at 95 °C for 15 sec, and annealing at 60 °C for 30 sec. The real-time RT-PCR was performed on the QuantStudio™ 7 Flex Real-Time PCR System and analyzed by the QuantStudio™ Real-Time PCR Software v1.3 (Thermo Fisher Scientific, Waltham, MA, USA). A no-template control (NTC) and a negative extraction control were used as negative controls.

### 2.5. Shotgun Metagenomics by Illumina and Bioinformatic Analysis

To obtain the complete sequence of the viral genome, the same cDNA sample loaded into the MinION platform was used for library preparation by the Nextera XT Library Prep Kit (Illumina Inc., San Diego, CA, USA). Deep sequencing was performed by the NextSeq 500 instrument (Illumina Inc.) using NextSeq 500/550Mid Output Reagent Cartridge v2 (Illumina Inc.), 300 cycles, and standard 150 bp paired-end reads. A *de novo* assembly was performed using SPAdes (version 3.11 [34,35]) based on multiple kmer lengths. 

### 2.6. Genome Characterization and Phylogeny 

Whole genome sequences representative of the genus *Mamastrovirus*, including sequences of AstVs connected with neurological disease reports, and of the genus *Avastrovirus* (as an outgroup rooting) were retrieved from Genbank and aligned with that obtained in this study using the MAFFT system [36]. Phylogenetic analysis of the whole genome was carried out using the Maximum Likelihood (ML) method implemented in MEGA version 6 [37]. The best-fit model of nucleotide (nt) substitution was identified using the Find Best DNA/protein model available in Mega 6 (GTR + G). To assess the robustness of individual nodes on the phylogenetic trees, a bootstrap resampling analysis with 500 replications was performed using default procedures available in MEGA6. The ORF2, coding for the capsid protein, was extracted from the full-length sequence alignment, translated, and re-aligned. This alignment was used to generate a phylogenetic tree using the Maximum Likelihood (ML) method implemented in MEGA version 6 [37] as described above, with the exception that as it is an amino acidic sequence, and the standard LG substitution model was used [38].

### 2.7. In Situ Investigation

For in situ detection of BoAstV PE3373/2019/Italy, formalin-fixed, paraffin-embedded (FFPE) tissue slides of the hippocampus, the medulla oblongata and the vermis were stained with chromogenic in situ hybridization (ISH). Chromogenic ISH was carried out with the RNAscope 2.5 Detection Kit—Brown (Advanced Cell Diagnostics, Newark, NJ, USA) and the probe against BoAstV CH13/NeuroS1 (Cat No. 406921), as described by the manufacturer. Slides were counter-stained with Mayers Hämalaunlösung (Merck KGaA, Darmstadt, Germany) and mounted with Aquatex^®^ (Merck KGaA, Darmstadt, Germany) mounting media before analyzing on a Zeiss Axio Scope.A1 (Carl Zeiss Microscopy GmbH, Göttingen, Germany) microscope. 

Double-staining with fluorescent ISH and immunofluorescence (IF) was performed on the FFPE slides. Therefore, firstly, ISH was performed using the RNAscope system (Advanced Cell Diagnostics, Newark, NJ) and the BoAstV CH13/NeuroS1 probe (Cat No. 406921). The assay was carried out with the RNAscope 2.5 Detection Kit—Red (Advanced Cell Diagnostics, Newark, NJ, USA), according to the manufacturer’s instructions, up to the step of signal detection. The protease pretreatment was slightly decreased to 20 min instead of 30 min so as not to harm the tissue too much for the following IF. All IF incubation steps were carried out in humid conditions. The slides were first washed in phosphate buffer saline containing 0.5% Tween (PBS-T) and blocked in 10% normal goat serum for 20 min at room temperature. Then, the BoAstV CH13 ORF2-con antibody [22] was incubated at 4 °C overnight. Afterwards, the slides were again washed with PBS-T and a secondary Alexa Fluor 488 goat anti-rabbit antibody (Abcam plc, Cambridge, UK) in a 1:1000 dilution, and DAPI BioChemica (AppliChem GmbH, Darmstadt, Germany) in a 1:10000 dilution were incubated for 1 h at room temperature. Finally, the slides were again washed in PBS-T and distillated water and mounted with Glycergel^®^, Aqueous Mounting Medium (Dako Denmark A/S, Glostrup, Denmark). Evaluation was done on an Olympus Fluoview FV3000 Confocal Laser Scanning Microscope (Olympus Europa, Hamburg, Germany). FFPE brain tissue slides of an animal with non-suppurative encephalitis but without BoAstV CH13 infection, as well as of the Swiss BoAstV CH13 index case (ID 45664, [16]) served as negative and positive controls, respectively.

## 3. Results

### 3.1. Major Neurotropic Pathogens Were Absent and Acute Non-Suppurative Encephalomyelitis Was Evidenced

**Figure 1:**
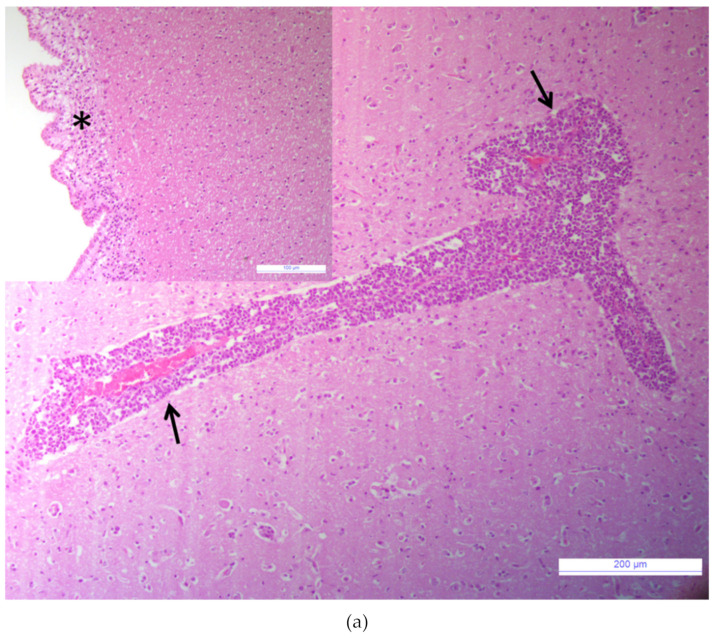

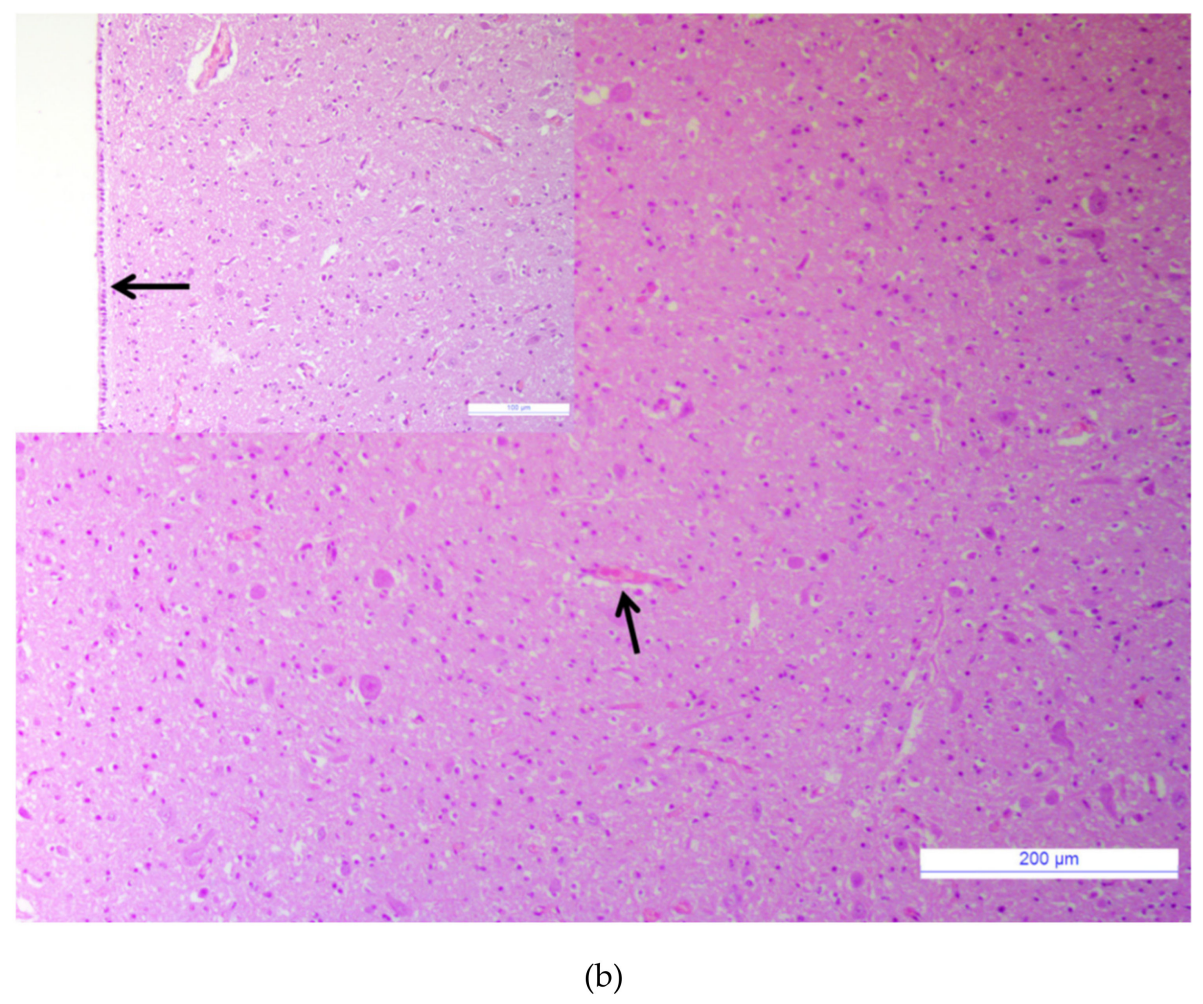
Brainstem. (**a**) Non-suppurative perivascular cuffing with many layers of lymphocytes, histiocytes, and plasma cells, (arrows), Hematoxylin and eosin (HE), bar: 200 µm. Inset: Brainstem. Sub ependymal oedema with infiltration of lymphocytes, (asterisk); HE, bar: 100 µm. (**b**) Brainstem of a healthy calf, normal blood vessel without inflammatory perivascular cuff (arrow), HE, bar: 200 µm. Inset: Brainstem. Normal ependymal cells layer, (arrow), HE, bar: 100 µm.

Samples tested negative for the major agents responsible for neurological signs in cattle. The brain tissue sample tested negative for the RNA/DNA of investigated viruses (BVDV, BoHV-1, OvHV-2, WNV, USUV), bacteria (*Chlamydia psittaci, Listeria spp.*), and parasites (*Neospora caninum, Toxoplasma gondii*). Bacterial growth was not observed when the brain homogenate was cultured with standard protocol. The histological examination revealed acute non-suppurative encephalomyelitis affecting the brainstem, cerebrum, cerebellum, and medulla oblongata. Diffuse perivascular cellular cuffs, characterized by 2 to 10 layers of lymphocytes, a few histiocytes, and plasma cells were present in all sections examined (Figure 1a). Multifocal infiltrates of lymphocytes and edema were present in the subependymal layers (Figure 1a, inset). Mild microgliosis and neuronal necrosis were also observed within some sections. Spongiform changes in the brain were absent. No lesions were observed in the control tissue (Figure 1b).

### 3.2. Bovine Astrovirus RNA Was Identified by MinION and Confirmed by RT-qPCR_BoAstV_

By assessing a quality cut-off (> Q7), a total number of 143,054 reads were obtained from the cDNA sequencing run with an average quality score of 924 and average length of 606 nt. Of these, 16,839 were classified as eukaryotes, 275 as bacteria, and 156 as viruses. The bacteria reads were identified as ubiquitous environmental bacteria. With reference to the virus reads, a total number of 151 were classified as BoAstV. The five remaining viral reads were assigned to three viruses, including *Avian coronavirus* (four reads) and *Leucania separate nucleopolyhedrovirus* (one read). From the DNA sequencing run, a total number of 707,347 reads were produced with an average quality score of 929 and average length of 634 nt. Of these, 65,393 were classified as eukaryotes, 3708 as bacteria, and 78 as viruses. Out of these viral reads, 18 were assigned to *Columbid alphaherpesvirus*, six to *Phaeocystis globosa* virus, and four to *Pandoravirus dulcis*.Also, in the DNA run, a batch of single reads assigned to different viruses was obtained.Importantly, the presence of RNA belonging to BoAstV was confirmed using the specific molecular assay (RT-qPCR_BoAstV_), displaying a cycle threshold of 25. 

### 3.3. BoAstV PE3373/2019/Italy Showed the Highest nt Sequence Identity with BoAstV CH13/NeuroS1

The run performed by the NextSeq 500 instrument produced a total number of 2,979,610 raw reads. Trimming was carried out using Trimmomatic software [39] with a cut-off for quality and a minimum length of reads fixed at a Phred score of 28 and 35 nt, respectively. After trimming, a total number of 2,543,994 paired and 209,847 unpaired reads were obtained. De novo assembly generated a single contig of BoAstV genomes of 6436 nt, which was used as a reference for remapping paired and unpaired reads by using Bowtie2 (version 2.3.4.1). Out of these, 1238 reads were mapped on the de novo contig with an average quality of 33.8, average length of 144 nt, and 28X of final coverage. The almost complete genome sequence of BoAstV PE3373/2019/Italy was deposited with the Genbank database (accession number MN464146). The obtained sequence lacked the entire 5’UTR region (51 nt), the first 9 nt of the ORF1a sequence, and the final 27 nt of the 3’UTR region. Genome organization was shown to be typical of the astrovirus, including three ORFs (ORF1a, ORF1b, and ORF2) with a ribosomal slippery sequence (5’-AAAAAAC-3’) between ORF1a and ORF1b, resulting in translation of the nonstructural polyprotein ORF1ab. The nucleotide sequence of BoAstV PE3373/2019/Italy showed the highest nt sequence identity (94.11%) with BoAstV CH13/NeuroS1 26730 (acc.no. KX266902). Using the blastp platform online, a comparison of protein sequences of nsp 1ab and the capsid polyprotein revealed the highest aa sequence identity (98.97% and 99.61%, respectively) with BoAstV CH13/NeuroS1 23871 (acc. no. KX266901) and BoAstV CH13/NeuroS1 36716 (acc. no. KX266904), respectively. Taking into account only the ORF1a coding sequence, BoAstV PE3373/2019/Italy showed a 98.83% aa identity with BoAstV CH13/NeuroS1 23871, 23985 (acc. no. KX266905), and 43660 (acc. no. KX266908) isolates. BoAstV CH13/NeuroS1 23871, BoAstV CH13/NeuroS1 23985, BoAstV CH13/NeuroS1 26730, BoAstV CH13/NeuroS1 36716, and BoAstV CH13/NeuroS1 43660 are BoAstV CH13 strains identified during a retrospective study and derived from frozen brain tissues of cattle diagnosed with AstV-positive encephalitis collected in Switzerland during the period from 1995 to 2009 [17].

### 3.4. BoAstV PE3373/2019/Italy Groups with BoAstV CH13/NeuroS1 Strains

**Figure 2:**
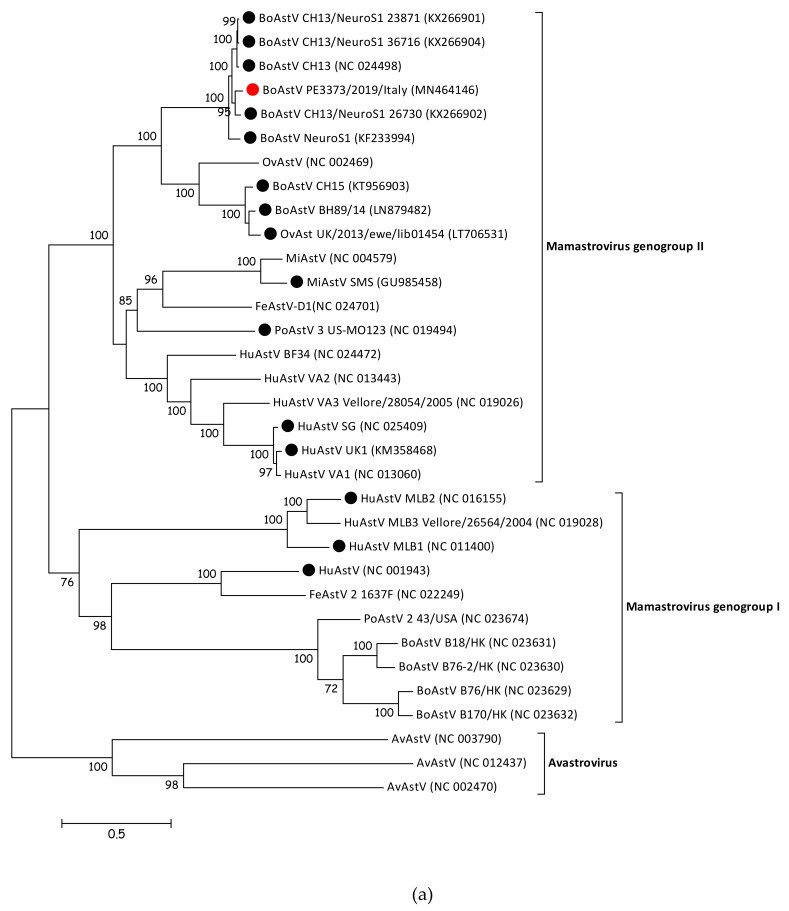

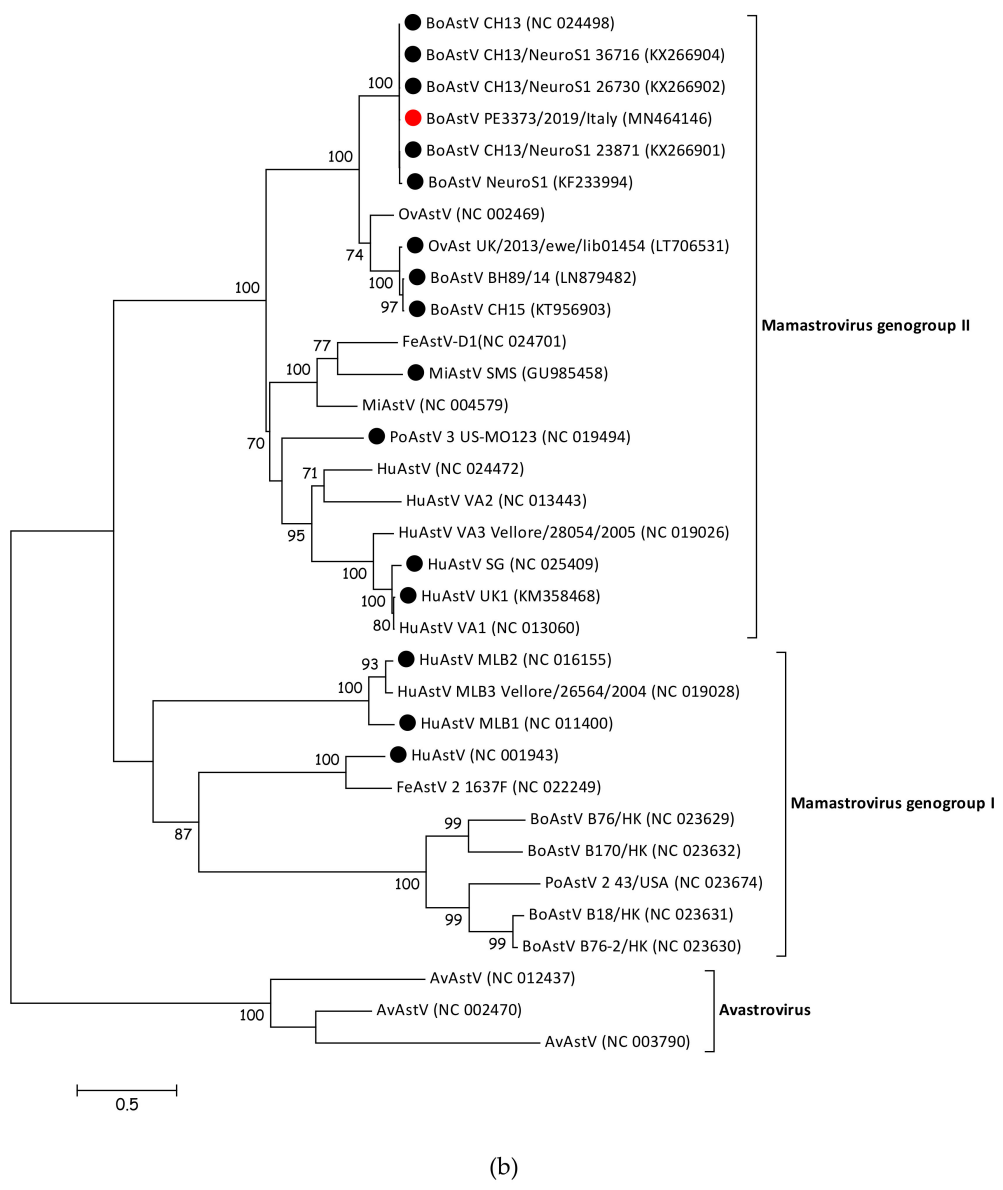
(**a**) Phylogenetic analysis of nucleotide sequence alignment of whole genome sequences of AstV representative members retrieved from GenBank and the BoAstV PE3373/2019/Italy detected in this study (identified with a red circle). Strains associated with encephalitis are identified with a black circle. Accession numbers are provided in brackets. Bar indicates the estimated numbers of nt substitutions per site. Bootstrap values ≥70 are shown. (**b**) Phylogenetic analysis of amino acid sequence alignment of the ORF 2 coding region of AstV representative members retrieved from GenBank and the BoAstV PE3373/2019/Italy detected in this study (identified with a red circle). Strains associated with encephalitis are identified with a black circle. Accession numbers are provided in brackets. The bar indicates the estimated numbers of aa substitutions per site. Bootstrap values ≥70 are shown. Sequences of both trees were retrieved from a previously published study [23].

To determine the phylogenetic relationship of BoAstV PE3373/2019/Italy to other members of the *Astroviridae* family, the sequence was compared with whole genome sequences of representative strains of *Mamastrovirus* and *Avastrovirus* genera. The resulting phylogenetic tree (Figure 2a) showed that members of *Avastrovirus* and *Mamastrovirus* genera are divided into two different clusters. Within the *Mamastrovirus* cluster, it was possible to identify two further groups, the genogroup I, containing classical strains, and the genogroup II, including strains associated with a diagnosed encephalitis (Figure 2a, identified with a black dot), as well as BoAstV PE3373/2019/Italy (Figure 2a, identified with a red dot). The previously observed similarities between the strain described in this manuscript and BoAstV CH13/NeuroS1 strains [17] were confirmed by phylogenetic analysis. BoAstV PE3373/2019/Italy clustered together with BoAstV CH13 [16], BoAstV NeuroS1 [15], and BoAstV CH13/NeuroS1 [17] strains, separated from the group, were formed by BoAstV CH15 [19], BoAstV BH89/14 [18], and OvAstV UK/2013/eke/lib01454 [23]. The phylogenetic analysis of the ORF2 gene region (Figure 2b), coding for the capsid protein, resulted in the same genogroup assignment as based on full-length sequences, grouping BoAstV PE3373/2019/Italy together with BoAstV CH13 [16], BoAstV NeuroS1 [15], and BoAstV CH13/NeuroS1 [17] strains within the *Mamastrovirus* genogroup II.

### 3.5. BoAstV PE3373/2019/Italy Presence in Cells Was Confirmed by ISH

**Figure 3:**
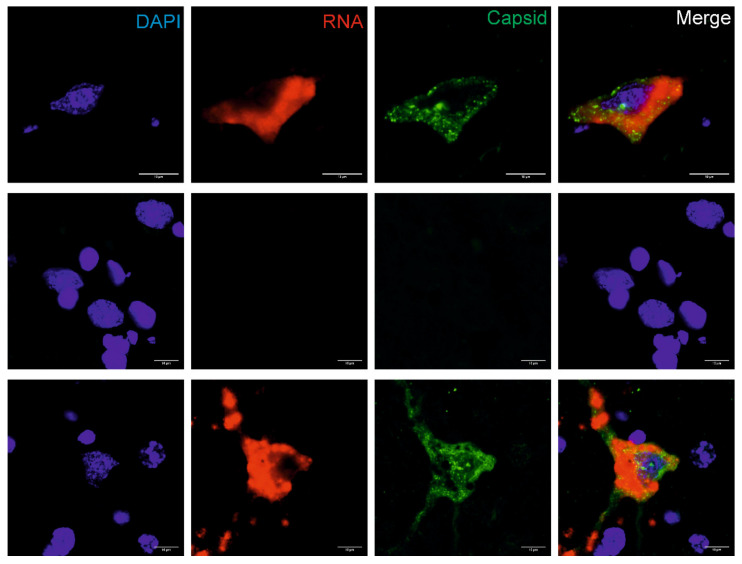
*In situ* detection of BoAstV CH13 in the hippocampus. Formalin-fixed, paraffin-embedded (FFPE) brain sections of the hippocampus of the BoAstV PE3373/2019/Italy case (top panel), a negative control animal (middle lane), and a BoAstV CH13 positive case (bottom panel) are presented. Cell nuclei were stained with DAPI (blue), viral RNA by fluorescent in situ hybridization (red), and the viral capsid protein by immunofluorescence (green). In the BoAstV PE3373/2019/Italy case and in the positive control, viral RNA and antigens co-localized the same cells (merged pictures), which suggests active replication of the virus. The negative control shows neither staining for viral RNA, nor for viral antigen. Microphotographs were taken at ×60 magnification.

The presence of BoAstV PE3373/2019/Italy RNA was confirmed by chromogenic ISH using a probe binding to the ORF2 region of the viral (+) genomic RNA (Figure S1). Positive staining consists of granular brown deposits, and could be seen in cells that were morphologically evaluated as neurons. With double-staining of viral RNA and antigens using the combination of fluorescent ISH and IF techniques, it was possible to detect BoAstV PE3373/2019/Italy RNA and antigen in situ in the same cells. Positive staining showed red (RNA) and/or granular green (antigen) fluorescent deposition, and was again found in neurons in all investigated brain areas. The highest level of positivity was observed in the hippocampus (Figure 3). Negative controls remained negative in all in situ assays, whereas the positive control revealed comparable signals. 

## 4. Discussion

Using a viral metagenomic approach, we identified a BoAstV strain, BoAstV PE3373/2019/Italy, in the brain of a heifer that presented with neurological symptoms. Brain samples tested negative for the agents responsible for neurological signs whose molecular tests were available in the diagnostic workflow at IZSAM. Clinical signs in this animal were consistent with previous reports on astrovirus-associated encephalitis in cattle [40]. By means of the MinION sequencing technology, we were able to rapidly identify the presence of BoAstV RNA and to rule out the presence of other pathogens. In this regard, the additional viral reads assigned to other viruses were not suggestive, as far as we know, of a plausible causal relationship with the observed clinical signs. Moreover, the presence of *Avian coronavirus* reads was likely related to the phenomenon known as cross-talk between barcoded samples [41], as a kidney sample of a chicken tested positive for infectious bronchitis virus (IBV, species *Avian coronavirus*) was loaded in the same run. Real-time RT-PCR and ISH confirmed the presence of BoAstV RNA in the brain. Using the combination of fluorescent ISH and IF techniques, it was possible to detect BoAstV RNA and antigens in the same cells, suggesting the active replication of the virus in infected neurons. Taken together, all these findings suggest a plausible causal relationship between BoAstV PE3373/2019/Italy and the observed neurological clinical signs. This result underscores the value and the versatility of MinION, supporting its potential for a rapid diagnosis when the possible etiologic agent identity is not found among the expected tested pathogens [27,30]. By using Illumina NextSeq 500, we also obtained nearly the whole genome sequence of BoAstV PE3373/2019/Italy. The sequence was closely related to BoAstV strains associated with non-suppurative encephalitis, identified in USA and Switzerland [15,16,17]. Although in mammalian species most strains were identified in fecal samples of healthy individuals or with enteric symptoms [42,43,44,45,46], in recent years an increasing number of neurotropic AstVs were identified in individuals with neurological clinical signs [10,15,16,18,19,20,22,38,47]. In particular, in ruminant species, neurotropic AstV strains are genetically closely related and it appears that they may share a common genetic ancestor (Figure 2), although the link between the capacity for neuronal invasion and neurotropism of these AstVs with their genetic features is yet to be demonstrated. Nevertheless, neurotropic BoAstVs appear to be remarkably conserved, including phylogenetically related sequences identified from 1995 to those in distinct geographic areas nowadays, and clearly different from previously described AstV species (genogroup I), in particular from those detected in fecal samples from healthy cattle with no enteric or other documented symptoms [48]. The strain identified in this study is unlikely to be epidemiologically related to those identified in Switzerland and USA. The likely neurotropic BoAstVs were already geographically widely spread but undetected and underestimated until recent studies. Accordingly, the number of AstV associated with encephalitis may also be much larger than currently known. Here, we provided further evidence of the role of AstV as a neurotropic pathogenic agent. Seroprevalence studies in healthy cattle should be taken to verify how frequently asymptomatic infections occur, maintaining the virus in the herd, whereas only some animals develop encephalitis. It seems that heifers in the age range of 1.5 to 2.5 years are particularly susceptible to the disease [16], as was the case described in this manuscript. Epidemiological links between AstV infections in animals and humans have been observed, and diverse novel human AstVs displayed close genetic relatedness to animal AstVs [42,49,50]. Indeed, phylogenetic features also suggest that neurotropic AstVs may share a common ancestor and, therefore, future research into astrovirus infections should include collaboration between veterinary and human health systems within the context of a One Health concept. Further studies should be aimed at investigating the mechanisms of the disease pathogenesis, identifying virus and host factors that contribute to the development of central nervous system infection, as well as the epidemiology of the neurotropic AstVs. New metagenomic approaches, such as the MinION technology here described, should be exploited in cases such as this one, since many cases of encephalitis of unknown etiology, both in humans and in animals, are still being reported. 

## Supplementary Materials

The following are available online at https://www.mdpi.com/1999-4915/12/5/530/s1, Figure S1: Detection of BoAstV CH13 RNA by chromogenic in situ hybridization in neurons of the hippocampus of the BoAstV PE3373/2019/Italy case (A, B), the positive control brain tissue (C) and the negative control brain tissue (D). Microphotographs were taken at ×40 magnification.

## References

[1] Matsui S.M., Greenber H.B., Felds B.N., Knipe D.M., Howely P.M., Chanock R.M., Melnick J.L., Monath T.P., Roizman B., Straus S.E. (1996). Astroviruses. Fields Virology.

[2] De Benedictis P., Schultz-Cherry S., Burnham A., Cattoli G. (2011). Astrovirus infections in humans and animals – Molecular biology, genetic diversity, and interspecies transmissions. Infect. Genet. Evol..

[3] Koci M.D., Schultz-Cherry S. (2002). Avian astroviruses. Avian Pathol..

[4] Bosch A., Pintó R.M., Guix S. (2014). Human Astroviruses. Clin. Microbiol. Rev..

[5] Lewis T.L., Matsui S.M. (1997). Studies of the astrovirus signal that induces (−1) ribosomal frameshifting. Adv. Exp. Med. Biol..

[6] Kiang D., Matsui S.M. (2002). Proteolytic processing of a human astrovirus nonstructural protein. J. Gen. Virol..

[7] Méndez E., Fernández-Luna T., López S., Méndez-Toss M., Arias C.F. (2002). Proteolytic Processing of a Serotype 8 Human Astrovirus ORF2 Polyprotein. J. Virol..

[8] Walter J.E., Mitchell D.K. (2003). Astrovirus infection in children. Curr. Opin. Infect. Dis..

[9] Appleton H., Higgins P.G. (1975). Letter: Viruses and gastroenteritis in infants. Lancet.

[10] Quan P.-L., Wagner T.A., Briese T., Torgerson T.R., Hornig M., Tashmukhamedova A., Firth C., Palacios G., Baisre-De-Leon A., Paddock C.D. (2010). Astrovirus Encephalitis in Boy with X-linked Agammaglobulinemia. Emerg. Infect. Dis..

[11] Brown J.R., Morfopoulou S., Hubb J., Emmett W.A., Ip W., Shah D., Brooks T., Paine S.M.L., Anderson G., Virasami A. (2015). Astrovirus VA1/HMO-C: An Increasingly Recognized Neurotropic Pathogen in Immunocompromised Patients. Clin. Infect. Dis..

[12] Frémond M.-L., Pérot P., Muth E., Cros G., Dumarest M., Mahlaoui N., Seilhean D., Desguerre I., Hébert C., Corre-Catelin N. (2015). Next-Generation Sequencing for Diagnosis and Tailored Therapy: A Case Report of Astrovirus-Associated Progressive Encephalitis. J. Pediatr. Infect. Dis. Soc..

[13] Naccache S.N., Peggs K.S., Mattes F.M., Phadke R., Garson J.A., Grant P., Samayoa E., Federman S., Miller S., Lunn M.P. (2015). Diagnosis of Neuroinvasive Astrovirus Infection in an Immunocompromised Adult With Encephalitis by Unbiased Next-Generation Sequencing. Clin. Infect. Dis..

[14] Lum S.H., Turner A., Guiver M., Bonney D., Martland T., Davies E., Newbould M., Brown J., Morfopoulou S., Breuer J. (2016). An emerging opportunistic infection: Fatal astrovirus (VA1/HMO-C) encephalitis in a pediatric stem cell transplant recipient. Transpl. Infect. Dis..

[15] Li L., Diab S., McGraw S., Barr B., Traslavina R., Higgins R., Talbot T., Blanchard P., Rimoldi G., Fahsbender E. (2013). Divergent Astrovirus Associated with Neurologic Disease in Cattle. Emerg. Infect. Dis..

[16] Bouzalas I.G., Wüthrich D., Walland J., Drögemüller C., Zurbriggen A., Vandevelde M., Oevermann A., Bruggmann R., Seuberlich T. (2014). Neurotropic Astrovirus in Cattle with Nonsuppurative Encephalitis in Europe. J. Clin. Microbiol..

[17] Bouzalas I.G., Wüthrich D., Selimovic-Hamza S., Drögemüller C., Bruggmann R., Seuberlich T. (2016). Full-genome based molecular characterization of encephalitis-associated bovine astroviruses. Infect. Genet. Evol..

[18] Schlottau K., Schulze C., Bilk S., Hanke D., Höper D., Beer M., Hoffmann B. (2016). Detection of a Novel Bovine Astrovirus in a Cow with Encephalitis. Transbound. Emerg. Dis..

[19] Seuberlich T., Wüthrich D., Selimovic-Hamza S., Drögemüller C., Oevermann A., Bruggmann R., Bouzalas I. (2016). Identification of a second encephalitis-associated astrovirus in cattle. Emerg. Microbes Infect..

[20] Blomström A.-L., Widén F., Hammer A.-S., Belák S., Berg M. (2010). Detection of a Novel Astrovirus in Brain Tissue of Mink Suffering from Shaking Mink Syndrome by Use of Viral Metagenomics. J. Clin. Microbiol..

[21] Boujon C.L., Koch M.C., Kauer R.V., Keller-Gautschi E., Hierweger M.M., Hoby S., Seuberlich T. (2019). Novel encephalomyelitis-associated astrovirus in a muskox (Ovibos moschatus): A surprise from the archives. Acta Vet. Scand..

[22] Boujon C.L., Koch M.C., Wüthrich D., Werder S., Jakupovic D., Bruggmann R., Seuberlich T. (2017). Indication of Cross-Species Transmission of Astrovirus Associated with Encephalitis in Sheep and Cattle. Emerg. Infect. Dis..

[23] Pfaff F., Schlottau K., Scholes S., Courtenay A., Hoffmann B., Höper D., Beer M. (2017). A novel astrovirus associated with encephalitis and ganglionitis in domestic sheep. Transbound. Emerg. Dis..

[24] Selimovic-Hamza S., Boujon C.L., Hilbe M., Oevermann A., Seuberlich T. (2017). Frequency and pathological phenotype of bovine astrovirus CH13/NeuroS1 infection in neurologically-diseased cattle: Towards assessment of causality. Viruses.

[25] Reuter G., Pankovics P., Boros A. (2018). Nonsuppurative (Aseptic) Meningoencephalomyelitis Associated with Neurovirulent Astrovirus Infections in Humans and Animals. Clin. Microbiol. Rev..

[26] Vandevelde M., Higgins R., Oevermann A. (2012). Veterinary Neuropathology: Essentials of Theory and Practice.

[27] Peserico A., Marcacci M., Malatesta D., Di Domenico M., Pratelli A., Mangone I., D’Alterio N., Pizzurro F., Cirone F., Zaccaria G. (2019). Diagnosis and characterization of canine distemper virus through sequencing by MinION nanopore technology. Sci. Rep..

[28] Del Amo J., Sotelo E., Fernández-Pinero J., Gallardo C., Llorente F., Agüero M., Jiménez-Clavero M.A. (2013). A novel quantitative multiplex real-time RT-PCR for the simultaneous detection and differentiation of West Nile virus lineages 1 and 2, and of Usutu virus. J. Virol. Methods.

[29] Cavrini F., Pepa M.E.D., Gaibani P., Pierro A.M., Rossini G., Landini M.P., Sambri V. (2011). A rapid and specific real-time RT-PCR assay to identify Usutu virus in human plasma, serum, and cerebrospinal fluid. J. Clin. Virol..

[30] Beato M.S., Marcacci M., Schiavon E., Bertocchi L., Di Domenico M., Peserico A., Mion M., Zaccaria G., Cavicchio L., Mangone I. (2018). Identification and genetic characterization of bovine enterovirus by combination of two next generation sequencing platforms. J. Virol. Methods.

[31] Marcacci M., De Luca E., Zaccaria G., Di Tommaso M., Mangone I., Aste G., Savini G., Boari A., Lorusso A. (2016). Genome characterization of feline morbillivirus from Italy. J. Virol. Methods.

[32] Marcacci M., Sant S., Mangone I., Goria M., Dondo A., Zoppi S., van Gennip R.G.P., Radaelli M.C., Cammà C., van Rijn P.A. (2018). One after the other: A novel Bluetongue virus strain related to Toggenburg virus detected in the Piedmont region (North-western Italy), extends the panel of novel atypical BTV strains. Transbound. Emerg. Dis..

[33] Lüthi R., Boujon C.L., Kauer R., Koch M.C., Bouzalas I.G., Seuberlich T. (2018). Accurate and precise real-time RT-PCR assays for the identification of astrovirus associated encephalitis in cattle. Sci. Rep..

[34] Nurk S., Bankevich A., Antipov D., Gurevich A.A., Korobeynikov A., Lapidus A., Prjibelski A.D., Pyshkin A., Sirotkin A., Sirotkin Y. (2013). Assembling single-cell genomes and mini-metagenomes from chimeric MDA products. J. Comput. Biol..

[35] Bankevich A., Nurk S., Antipov D., Gurevich A.A., Dvorkin M., Kulikov A.S., Lesin V.M., Nikolenko S.I., Pham S., Prjibelski A.D. (2012). SPAdes: A New Genome Assembly Algorithm and Its Applications to Single-Cell Sequencing. J. Comput. Biol..

[36] Katoh K., Standley D.M. (2013). MAFFT Multiple Sequence Alignment Software Version 7: Improvements in Performance and Usability. Mol. Biol. Evol..

[37] Tamura K., Stecher G., Peterson D., Filipski A., Kumar S. (2013). MEGA6: Molecular Evolutionary Genetics Analysis Version 6.0. Mol. Biol. Evol..

[38] Guindon S., Gascuel O. (2003). A Simple, Fast, and Accurate Algorithm to Estimate Large Phylogenies by Maximum Likelihood. Syst. Biol..

[39] Bolger A.M., Lohse M., Usadel B. (2014). Trimmomatic: A flexible trimmer for Illumina sequence data. Bioinformatics.

[40] Deiss R., Selimovic-Hamza S., Seuberlich T., Meylan M. (2017). Neurologic Clinical Signs in Cattle with Astrovirus-Associated Encephalitis. J. Vet. Intern. Med..

[41] Xu Y., Lewandowski K., Lumley S., Pullan S., Vipond R., Carroll M., Foster D., Matthews P.C., Peto T., Crook D. (2018). Detection of Viral Pathogens with Multiplex Nanopore MinION Sequencing: Be Careful with Cross-Talk. Front. Microbiol..

[42] Bányai K., Meleg E., Moschidou P., Martella V. (2010). Detection of Newly Described Astrovirus MLB1 in Stool Samples from Children. Emerg. Infect. Dis..

[43] Martella V., Moschidou P., Pinto P., Catella C., Desario C., Larocca V., Circella E., Bànyai K., Lavazza A., Magistrali C. (2011). Astroviruses in Rabbits. Emerg. Infect. Dis..

[44] Martella V., Moschidou P., Lorusso E., Mari V., Camero M., Bellacicco A., Losurdo M., Pinto P., Desario C., Bányai K. (2011). Detection and characterization of canine astroviruses. J. Gen. Virol..

[45] Martella V., Moschidou P., Catella C., Larocca V., Pinto P., Losurdo M., Corrente M., Lorusso E., Bànyai K., Decaro N. (2012). Enteric Disease in Dogs Naturally Infected by a Novel Canine Astrovirus. J. Clin. Microbiol..

[46] Martella V., Decaro N., Buonavoglia C. (2015). Enteric viral infections in lambs or kids. Vet. Microbiol..

[47] Jiang H., Holtz L.R., Bauer I., Franz C.J., Zhao G., Bodhidatta L., Shrestha S.K., Kang G., Wang D. (2013). Comparison of novel MLB-clade, VA-clade and classic human astroviruses highlights constrained evolution of the classic human astrovirus nonstructural genes. Virology.

[48] Tse H., Chan W.-M., Tsoi H.-W., Fan R.Y.Y., Lau C.C.Y., Lau S.K.P., Woo P.C.Y., Yuen K.-Y. (2011). Rediscovery and genomic characterization of bovine astroviruses. J. Gen. Virol..

[49] Finkbeiner S.R., Le B.-M., Holtz L.R., Storch G.A., Wang D. (2009). Detection of Newly Described Astrovirus MLB1 in Stool Samples from Children. Emerg. Infect. Dis..

[50] Kapoor A., Li L., Victoria J., Oderinde B., Mason C., Pandey P., Zaidi S.Z., Delwart E. (2009). Multiple novel astrovirus species in human stool. J. Gen. Virol..

