# Supplementary material for: Detection of Astrovirus in a Cow with Neurological Signs by Nanopore Technology, Italy
Source: Viruses. 2020 May 11;12(5):530. doi: 10.3390/v12050530 (PMC7290991; doi:10.3390/v12050530)
Supplement: Supplementary file 1 [file viruses-12-00530-s001.pdf]

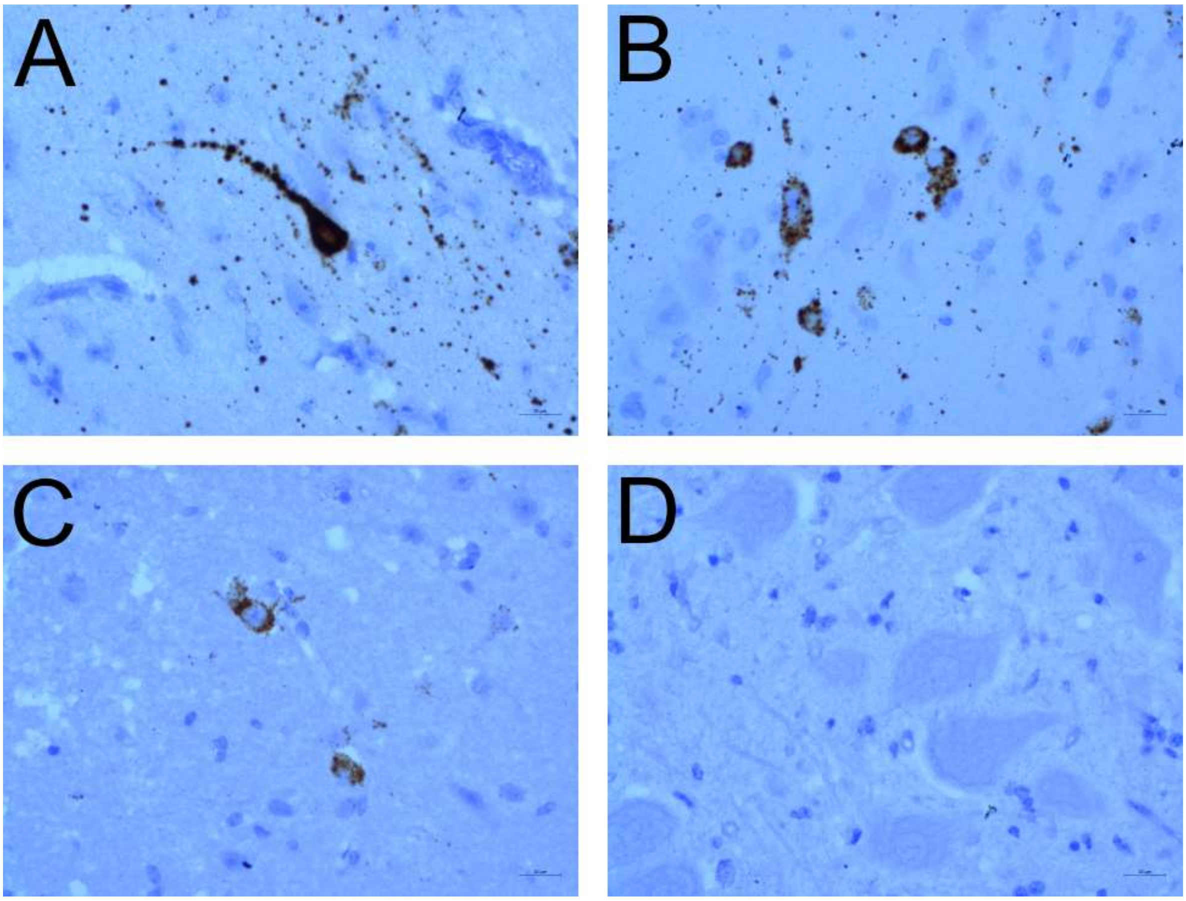


**Figure S1.** Detection of BoAstV CH13 RNA by chromogenic in-situ hybridization in neurons of the hippocampus of the BoAstV PE3373/2019/Italy case (A, B), the positive control brain tissue (C) and the negative control brain tissue (D). Microphotographs were taken at x40 magnification.
